# Explaining the heterogeneity in average costs per HIV/AIDS patient in Nigeria: The role of supply-side and service delivery characteristics

**DOI:** 10.1371/journal.pone.0194305

**Published:** 2018-05-02

**Authors:** Sergio Bautista-Arredondo, M. Arantxa Colchero, Ogbonna O. Amanze, Gina La Hera-Fuentes, Omar Silverman-Retana, David Contreras-Loya, Gregory A. Ashefor, Kayode M. Ogungbemi

**Affiliations:** 1 Center for Health Systems Research, Instituto Nacional de Salud Pública, Cuernavaca, Mexico; 2 UC, Berkeley. School of Public Health, Berkeley, California, United States of America; 3 National Agency for the Control of AIDS, Abuja, Nigeria; 4 Department of Public Health, Aarhus University, Aarhus, Denmark; Universidad Loyola Andalucia, SPAIN

## Abstract

**Objective:**

We estimated the average annual cost per patient of ART per facility (unit cost) in Nigeria, described the variation in costs across facilities, and identified factors associated with this variation.

**Methods:**

We used facility-level data of 80 facilities in Nigeria, collected between December 2014 and May 2015. We estimated unit costs at each facility as the ratio of total costs (the sum of costs of staff, recurrent inputs and services, capital, training, laboratory tests, and antiretroviral and TB treatment drugs) divided by the annual number of patients. We applied linear regressions to estimate factors associated with ART cost per patient.

**Results:**

The unit ART cost in Nigeria was $157 USD nationally and the facility-level mean was $231 USD. The study found a wide variability in unit costs across facilities. Variations in costs were explained by number of patients, level of care, task shifting (shifting tasks from doctors to less specialized staff, mainly nurses, to provide ART) and provider´s competence. The study illuminated the potentially important role that management practices can play in improving the efficiency of ART services.

**Conclusions:**

Our study identifies characteristics of services associated with the most efficient implementation of ART services in Nigeria. These results will help design efficient program scale-up to deliver comprehensive HIV services in Nigeria by distinguishing features linked to lower unit costs.

## Introduction

HIV prevalence in Nigeria was estimated at 3.2% in 2012, with prevalence among states ranging from 0.2% in Ekiti to 15.2% in Rivers [1]. In 2006, the government implemented a free antiretroviral treatment (ART) program and began to scale up access nation-wide, reaching 813 sites in 36 states by 2013, with 639,837 patients receiving treatment [1]. Currently, ART services are provided predominantly in tertiary and secondary level facilities and in few primary level facilities. Nigeria has implemented the largest antiretroviral treatment program in Africa. However, critical HIV financing shortfalls represent a significant barrier for further scale-up [1]. Improving efficiency without compromising quality in light of current financial challenges is therefore an essential implementation priority.

One approach to understand efficiency gaps and identify opportunities for improving implementation is to measure and analyze economic costs of services. However, only estimating the average cost per patient, or unit cost, is not sufficient; assessing and understanding variation of unit costs across facilities and identifying determinants of such variation offers an opportunity to learn important lessons. The hypothesis behind this approach is that heterogeneity in efficiency is not entirely random, and the larger the variation across facilities in terms of the costs per patient-year, the more important it is to understand it. Evidence suggests that in the case of HIV services, this type of variation is significantly large [2–5]. By studying factors associated with unit cost variation, we can learn from the most and least efficient providers and improve the overall facility-level performance.

Although Nigeria implements one of the largest ART programs in Africa [6], few ART costing studies have been conducted in this country to date. Menzies et al. conducted an HIV costing study in 6 countries in Africa (including Nigeria) and Asia, with a sample size of 9 sites per country to assess the facility-level and country determinants of costs (excluding antiretroviral drugs because there was no variation in prices) [3]. Aliyu et al. estimated the ART annual average cost per patient in two tertiary and seven secondary facilities in six regions in Nigeria [7]. This study analysed costs composition and cost variation across regions, facility type and location (urban/rural). Finally, a report in 2004 used data collected from five hospitals to estimate the annual cost per patient and costs composition (drugs, monitoring, human resources, capital and training) in Nigeria [8]. While these studies provide an overview of the costs of providing ART in Nigeria, they are based on small and non-representative samples of facilities. Furthermore, costs composition and determinants of cost variation were addressed only in one study [3].

The objectives of this study, thus, are 1) to estimate the average annual cost per patient on ART (unit cost) per facility in Nigeria, and 2) to describe the variation in costs across facilities and identify factors associated with this variation. We are particularly interested in exploring the relationship between cost variation and supply-side and service delivery model characteristics. Our study is the first to rely on a relatively large and representative sample of facilities in Nigeria.

## Methods

The Institutional Review Board (Ethics Committee) at the National Institute of Public Health in Mexico (IPF Code 3627801) approved the study as well as the Nigerian Institute for Medical Research Institutional Review Board. Before any provider or patient was interviewed at a facility, enumerators had to read a written informed consent describing the objectives of the study, what his/her participation involved, that participation was entirely voluntary and responses confidential. All participants willing to participate in the study signed the consent.

### Study sample

This study is part of a broader project named “Optimizing the Response of Prevention and Treatment: HIV Efficiency in Nigeria” (ORPTHEN). The objective of the ORPTHEN project was to estimate total costs and average costs per patient/client per facility and assess levels and determinants of efficiency for three HIV services: HIV counseling and testing (HCT), prevention of mother to child transmission (PMTCT), and antiretroviral treatment (ART). Our study focuses on the latter intervention.

We used a multi-stage selection process to identify 200 health facilities across the 17 Nigerian states with the highest HIV prevalence. In the first stage, we identified the 20 states with the highest HIV prevalence. Three high-prevalence states (Yobe, Adamawa, and Borno) were excluded due to security reasons. Although the remaining states share a similar overall HIV burden, they represent quite diverse contexts: Lagos and Kano comprise large metropolitan areas; Adamawa comprises relatively small urban localities; and Ondo is characterized by an agricultural economy and rural localities.

**Fig 1:**
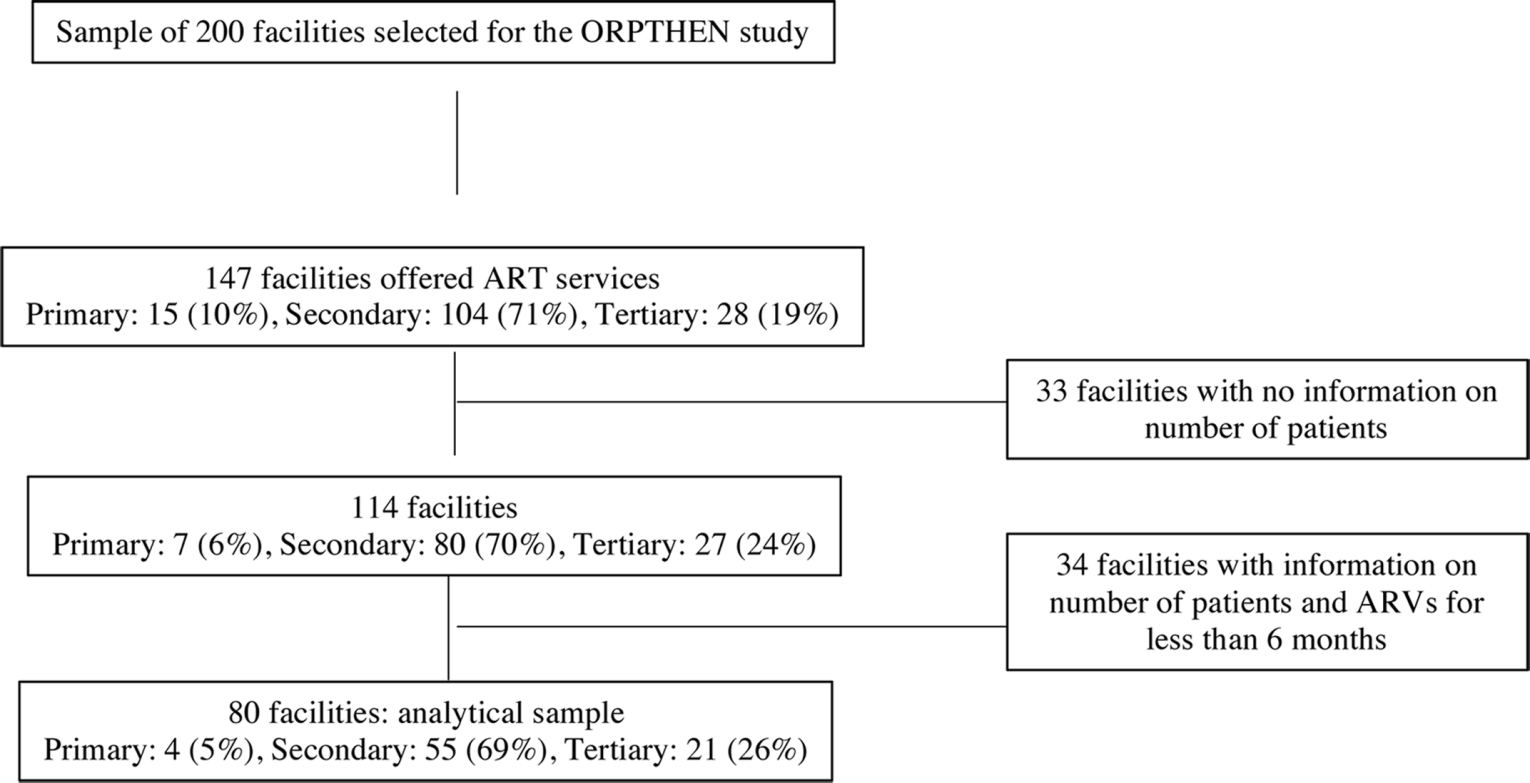
Analytical sample.

In the second stage, we selected a random sample of facilities providing HIV services in the primary, secondary and tertiary levels of care. As shown in Fig 1, of the 200 facilities included in the sample, 147 (73.5%) facilities offered ART services at the time of data collection (15 primary-level facilities, 104 secondary-level and 28 tertiary-level facilities). We further excluded 67 facilities due to missing or incomplete data; 33 facilities had no data on number of patients, and 34 had less than six months of data on ARV drugs and number of patients. The analytical sample (N = 80) excluded 11 primary-level facilities, 49 facilities at the secondary level and 7 tertiary-level hospitals. Descriptive analysis comparing the 67 excluded facilities to the analytical sample showed significant differences in time since they started offering ART services (39.5 months on average in the excluded sample, and 67.2 months in the analytical sample). Moreover, 35.8% of excluded facilities had less than 12 months offering ART services compared to 7.5% in the analytical sample (S1 Table). Thus, the analytical sample over represents more established facilities, which may have biased our results if this characteristic is systematically associated with costs. However, this bias is probably not relevant in the long term, as it is expected that all facilities become established at some point in time.

### Data collection

Data collection took place over six months between December 2014 and May 2015. Data on inputs, input prices, outputs, process quality, staff’s time allocation and facility-level characteristics, including management practices, were collected for the entire fiscal year 2013 using standardized, pre-programmed, computer-based instruments. Data collection followed a previously developed protocol (ORPHEA, a study to assess costs, cost variability and determinants of efficiency for HIV prevention interventions in Kenya, Rwanda, South Africa and Zambia) [9]. Teams of three data collectors were trained on the content and use of the instruments, the logistic procedures to follow in the field, and the voluntary and confidential character of data collection. Data were collected over a period of two to four days in the facility from different sources and informants, as described below. A data quality assurance system was simultaneously implemented, through which the research team based in Mexico downloaded the collected data on a weekly basis to evaluate data quality and data completeness in order to provide feedback to the data collection teams.

### Measurement

Data were collected retrospectively for each month of the entire previous fiscal year, corresponding to the following categories of inputs: titles and positions of all medical and non-medical staff involved in the provision of ART services, antiretroviral drugs dispensed over the year, capital and buildings, and time allocated to training and supervision activities. Laboratory tests (CD4) performed in 2013 were collected from clinical files in each facility. Viral loads are rarely used in Nigeria; therefore, we did not include costs of this type of test in our calculations.

Input prices for all inputs (salaries, ARVs, laboratory tests) were collected from the National Agency for the Control of AIDS. At the facilities, we also collected monthly expenditures on utilities and other recurrent inputs and services such as electricity, water, building maintenance, telephone, transportation and medical supplies. Outputs were measured from logs kept by the clinical staff and included monthly number of new and total pre-ART and ART patients.

We measured process quality through clinical vignettes [10–14]. The questionnaire was based on National Treatment Guidelines [15] valid at the time of data collection and included questions related to procedures for pre-ART and ART and TB co-infected patients. Procedures included laboratory test requested, clinical evaluation, and appointments scheduled for monitoring during a patient’s first and follow-up visits. Five doctors were interviewed in each facility, all randomly selected.

Data on time allocation of providers involved in ART services per facility were collected through interviews with providers. We randomly selected six providers for an interview to ask them about the time spent on activities performed during the last week of work and the services to which those activities corresponded–ART, other HIV services, non-HIV services, breaks, meetings or administrative work.

Staff in charge at the facilities were asked questions on managerial characteristics of the facility and their programs. More specifically, they were asked to identify management practices, grouped in six categories, that were routinely implemented in their facilities. The categories are: performance-based incentives, sanctions for poor performance, levels of external supervision received, transparency practices, and level of community involvement. The questions asked are presented in S2 Table in the supplemental material.

**Fig 2:**
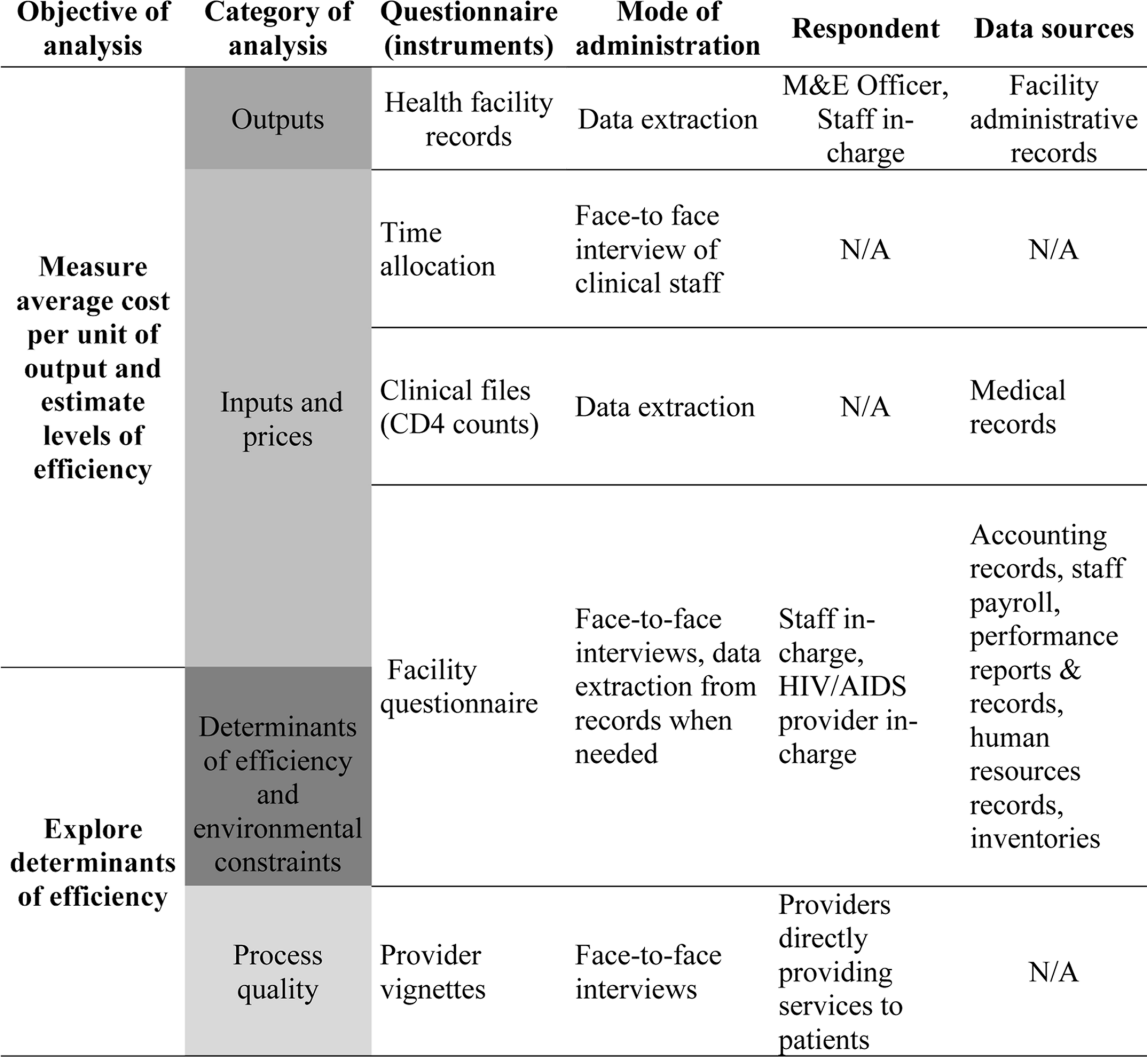
ORPTHEN questionnaires and data sources for different types of information.

All measurements were adapted from the methods of the ORPHEA project [9]. Fig 2 provides an overview of the measurement instruments (questionnaires) used to collect data, as well as the mode of administration, the informant(s), and data sources consulted. The figure also links these categories with the objectives and categories of analysis.

### Estimation of unit costs and country-level weighted annual cost per patient

Unit costs were estimated at the facility level by adding annual costs of staff, utilities, capital, training, laboratory tests (CD4), antiretroviral drugs (ARVs) and tuberculosis (TB) treatment drugs, divided by the number of pre-ART and ART patients reported in 2013, as follows:

$$UC_{j}=\frac{\sum_{i=1}^{i=6}IC_{ij}}{P_{j}}$$

where UC represents the unit cost at facility *j*. The term IC_*ij*_ represents the total annual cost of input category *i* at facility *j*, for input categories: 1: staff, 2: recurrent inputs and services, 3: capital, 4: training, 5: CD4 tests, and 6: ARVs and TB treatment drugs. *P*_*j*_ represents the annual number of ART and pre-ART patients at facility *j*. ARVs equals the average annual cost of ARVs per patient, multiplied by the number of patients on ART. TB treatment is the estimated TB treatment cost per patient, multiplied by the number of patients on TB treatment.

Some recurrent inputs and services, and most capital and training costs, are shared by different services. To estimate the share of these resources allocated to ART, we weighted the total costs of each input category by the relative workload attributable to ART. These weights are the proportion of ART patients over all patients at the facility. For shared providers, staff costs were estimated based on the proportion of time dedicated to ART activities as determined by the time allocation measurements. In the case of staff fully dedicated to ART, we allocated 100% of their time to ART.

The number of pre-ART and ART patients were obtained from monthly reports of new patients and total patients enrolled in HIV care. As patients visit the facilities several times in a year, these monthly figures for cumulative patients include repeated patients over time. To estimate the number of patients over the costing year, we summed patients under first and second line treatment reported monthly and then divided by 12 to get the annual average and added all pre-ART newly enrolled or transferred in from another facility over the year as follows:

$$P_{j}=\frac{\sum_{k=1}^{k=12}{PT}_{kj}}{12}+\sum_{k=1}^{12}{Pre}_{kj}$$

where P are patients at facility j, PT are ART patients on first and second line treatment at month k in facility j, Pre are pre-ART patients at month k in facility j (newly enrolled or transferred in).

We estimated the annual cost of ARVs per patient as the average price of all treatment regimens available at each facility as follows:

$${AP}_{j}=\frac{\sum_{i=1}^{i=n}{TR}_{ij}\;*\;{Price}_{i}}{R_{j}}*\;12$$

where AP_*j*_ denotes the average annual ARV cost per patient at a facility *j*, TR_*ij*_ denotes the treatment regimen *i* available at each facility *j* at any time during the costing year, *Price*_*i*_ is the monthly price of regimen i, and *R*_*j*_ is the number of regimens in facility *j*. Each treatment regimen corresponds to a monthly prescription; therefore, the average cost of all treatment regimens is multiplied by 12 months to obtain the annual cost per patient. This result was then multiplied by the annual number of ART patients at each facility to obtain the total annual cost on ARVs per facility.

ARV drugs included first line and second line regimens. Drug presentation was available on single tablet regimens (AZT+3TC+NVP, AZT+3TC+EFV, TDF+3TC+EFV, TDF+FTC+EFV), dual ARV presentation (AZT+3TC, TDF+3TC, TDF+FTC), as well as single ARV drugs for multi-tablet regimens (EFV, NVP, ABC, ATVr, LPVr). All regimens included in the estimation were partially imputed. Missingness occurred in cases in which data reported on individual drugs corresponded to incomplete regimens. In such cases, we imputed the missing element of the combination by estimating the number of bottles needed to fulfill a complete dose. The annual TB cost per patient was calculated using the same formula as for the annual ARV cost per patient. We used a similar imputation method for TB treatments.

For laboratory tests, we collected basic data on tests performed from a random sample of clinical files. From the subsample of facilities from which we had usable data from at least 20 files, we estimated the average number of CD4 count tests performed per year, per type of facility (primary, secondary or tertiary level). The resulting mean CD4 counts performed per year were: 1 for primary level, 1.5 for secondary level and 2 for tertiary level facilities. We extrapolated the average cost for CD4 count tests, by type of facility, to all facilities based on these results.

Finally, we estimated a national average annual ART cost per patient in Nigeria as the total ART costs divided by the total number of patients across all facilities:

$$ACP=\frac{\sum_{j}{Total\;costs}_{j}}{\sum_{j}P_{j}}$$

where total costs are defined as follows: ${Total\;costs}_{j}=\sum_{i=1}^{i=6}{IC}_{i,j}$

The resulting figure is a better estimate of the average cost of a patient in Nigeria, as opposed to the average unit cost, which is a representation of the average cost per patient per facility.

All monetary figures are presented in US dollar amounts. Currency amounts originally in Nigerian Naira (NGN) were converted into US dollar amounts using the annual exchange rate at which commercial banks were exchanging the two currencies (150 NGN = 1US$).

### Analysis of unit costs

#### Composition of unit costs

We estimated the distribution of total ART costs by component: staff, laboratory tests, ARV drugs, TB drugs, capital, utilities and training; and the distribution of total staff costs by type of staff: nurses, doctors, health and indirect staff.

#### Factors associated with facility-level variation of ART costs

We first explored the association between unit cost and scale (number of patients) using a locally weighted scatterplot smoothing to test for nonlinear associations. We looked at the correlation on a log-log scale differentiating by level of care (we grouped primary and secondary level facilities together and tertiary as a single group).

We then analyzed the factors associated with unit costs through an ordinary least squares (OLS) regression model with robust standard errors. As the distribution of costs was skewed and not normally distributed, we used the logarithm of the unit cost. The independent variables included in the models are listed in Table 1. These variables measure the following characteristics at the facility-level variables: supply-side characteristics, process quality, service delivery model, health providers´ experience and management practices. Through this model specification, we tested the hypothesis that management characteristics are directly associated with unit cost variations. Questions included in each management dimension are listed in S2 Table. We added an interaction term between number of patients and level of care to allow for non-linearities in the association between number of patients and unit cost, by level of care. For these regression models, we show results for a sample restricted to 63 facilities with available information from the health provider vignettes and health providers´ experience variables, in addition to showing the regression with the full analytical sample (N = 80) excluding these variables.

**Table 1:** Independent variables included in the analysis.

Dimension	Variable
Characteristics of the supply	• Annual number of patients (logarithm) • Level of care–a binary variable which takes the value 1 if tertiary level, and 0 if primary/secondary grouped together as there are only 4 primary level facilities-
*Process quality (competence)*	• Proportion of health providers with a competence score greater than or equal to 80% from the clinical vignettes
*Service delivery model*	• Task shifting–a binary variable which equals 1 if the proportion of doctors with respect to all health staff providing ART treatment is 0, and 1 otherwise
*Health providers´ experience*	• Proportion of health providers with a university degree or higher • Number of patients received per day • Providers with more than 10 years of experience -a continuous variable for number of providers
*Management*	Binary variables based on additive scores for the following measures of management practices: • Performance-based incentives (equal 1 if higher than the 75 percentile, 0 otherwise) • Incentives for good performance (equal 1 if higher than the 75 percentile, 0 otherwise) • Sanctions for poor performance (equal 1 if higher than the 25 percentile, 0 otherwise) • External supervisions received (equal 1 if higher than the median, 0 otherwise) • Transparency (equal 1 if higher than the 75 percentile, 0 otherwise) • Community involvement (equal 1 if higher than the 75 percentile, 0 otherwise)

In a second model specification, we tested an alternative hypothesis on the link between management and costs; namely, we tested the hypothesis that management practices do not directly determine cost variation, but rather modify the effect of other determinants of costs. Specifically, we modeled the association between costs and facility characteristics (scale, level of care, and service delivery model) by management dimension, using a fully interacted model in which we included interactions between all facility characteristics and the binary variables for management. Each management dimension was dichotomized by level of practice–high or low.

## Results

**Fig 3:**
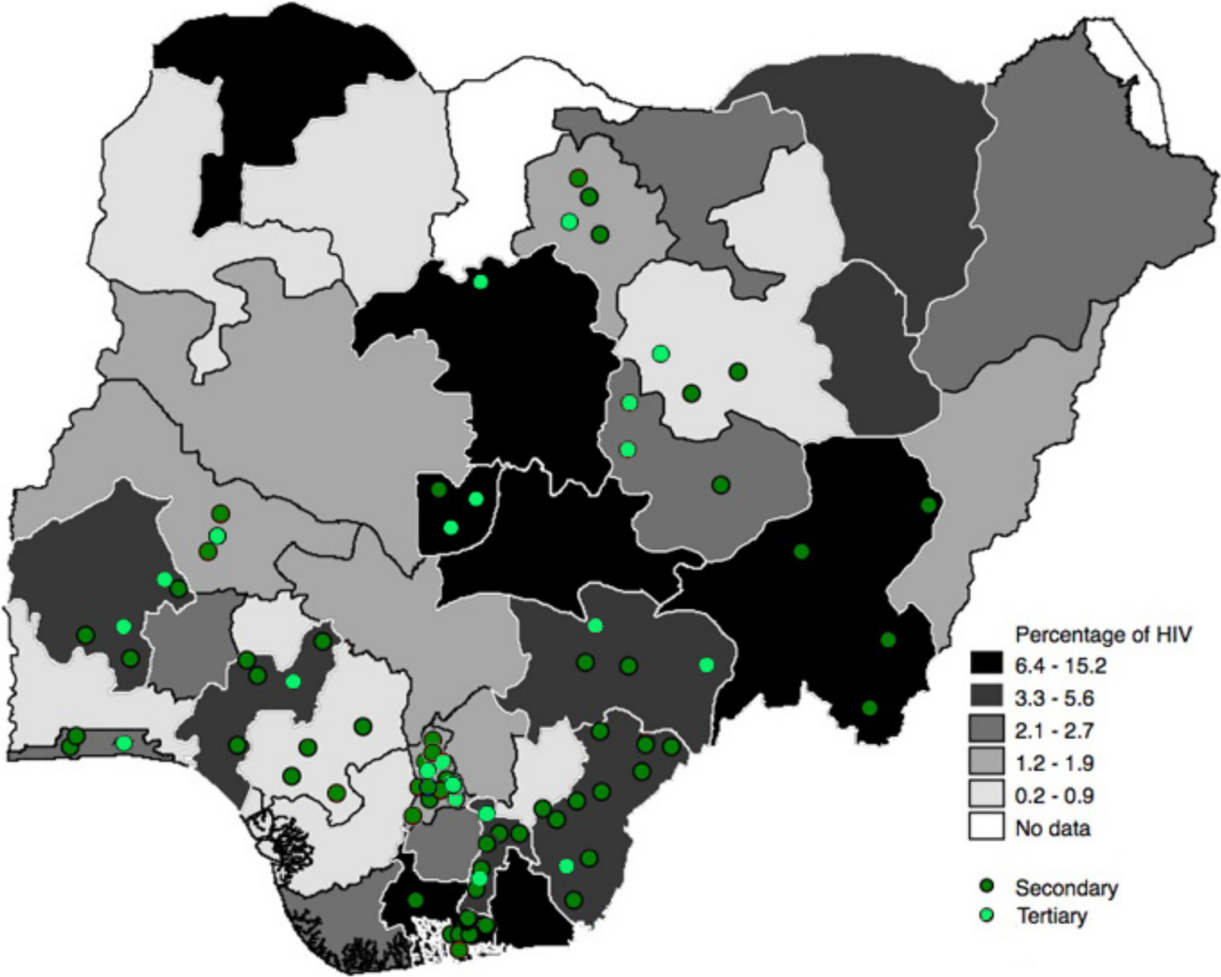
Geographic distributions of facilities and HIV prevalence by state.

Fig 3 depicts the map of Nigeria with state-level HIV prevalence and the sample of facilities included in the study by level of care.

Table 2 displays the distribution of the 80 facilities by level of care and ownership (public, private or faith-based) and the number of patients by ownership. The sample is distributed as follows: 4 primary care facilities (5%), 55 secondary-level facilities (69%) and 21 tertiary-level facilities (26%). Sixty-six percent of facilities were public. The sample includes 1,805 total ART patients 1,433 on ART and 372 pre-ART.

**Table 2:** Number of facilities and number of patients by ownership and level of care.

	Total	Public (66%)	Private or faith-based (34%)
**Level of care**			
Primary	4 (5%)	2 (4%)	2 (7%)
Secondary	55 (69%)	36 (68%)	19 (70%)
Tertiary	21 (26%)	15 (28%)	6 (23%)
Total	80 (100%)	53 (100%)	27 (100%)
**Patients (average)**			
ART	1,433	1,668	973
Pre-ART	372	428	261
Total	1,805	2,096	1,234
**TB comorbidity (average)**			
ART patients with TB	18	24	8
Pre-ART patients with TB	19	25	9
Total	44	57	19

The estimated annual average ART cost per patient was $231 USD and $334 USD adjusted for purchase power parity (PPP) (Table 3). The national average cost was estimated at $157 USD ($182 USD in PPP).

**Table 3:** Annual ART cost per patient in Nigeria across 80 facilities.

	Cost in US dollars	Cost in US dollars (PPP)*
**Mean**	**231**	**334**
Standard deviation	215	384
Minimum	71	81
25^th^ percentile	125	151
**Median**	**159**	**192**
75^th^ percentile	230	329
Maximum	1,612	2,783
National mean**	**157**	**182**

*Purchase power parity

**Weighted by a factor based on the number of patients each facility contributes to the entire sample

**Fig 4:**
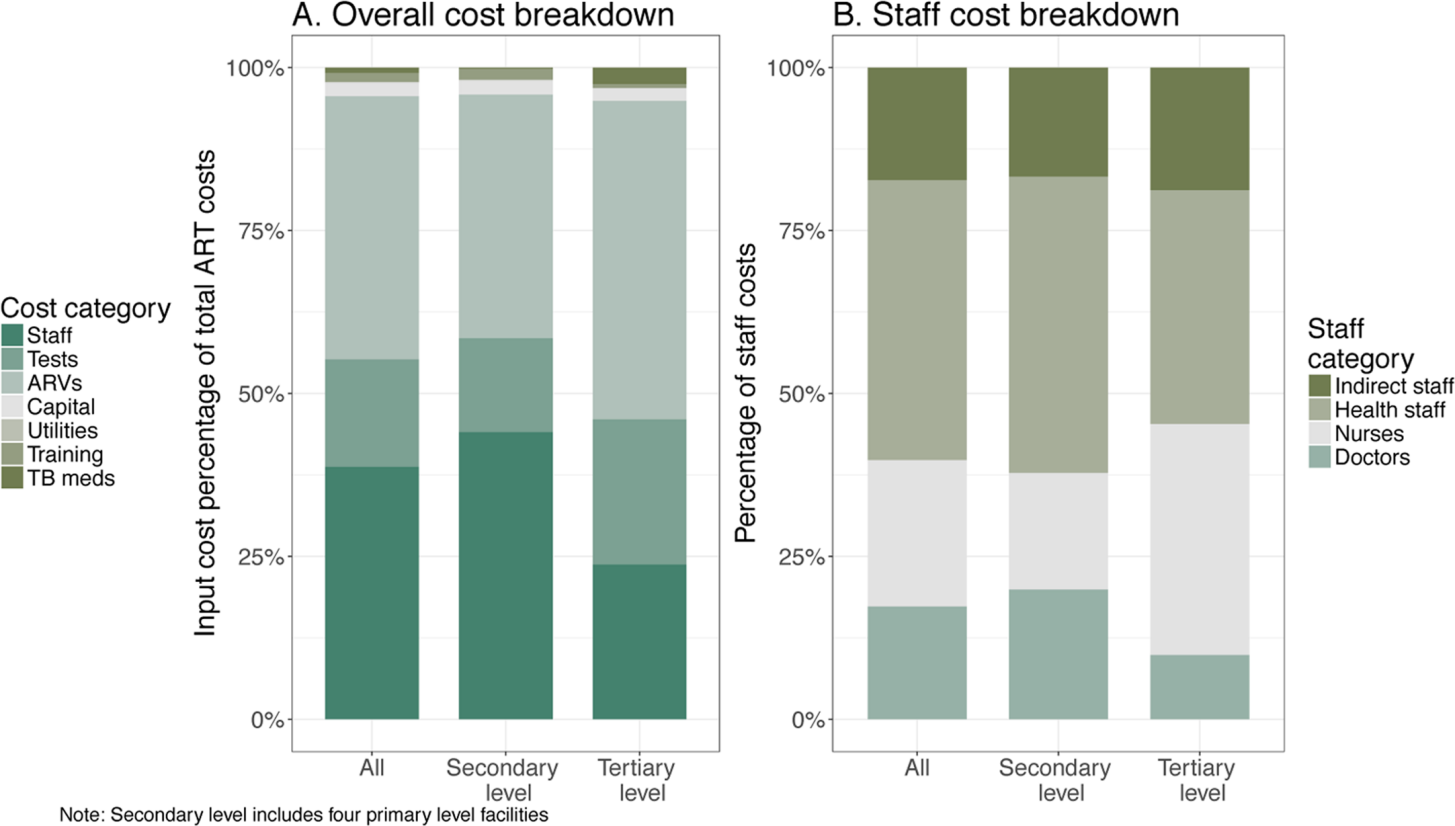
Total ART costs and staff cost breakdown.

As illustrated in Fig 4, staff, ARVs and laboratory tests are the largest components of the unit cost. For the overall sample, ARVs represent 40% of total costs; staff represents 39%; and laboratory tests account for 17%. The relative weight of ART input costs, however, varies by facility type. Staff costs represented a lower portion of total costs in tertiary level compared to secondary level facilities.

Fig 4 also shows the distribution of staff costs. For the overall sample, nurses and doctors represent 40% of total staff costs, other health staff (ART personnel in direct contact with patients, such as counselors, medical lab personnel, among others) represented 43%, and a smaller portion (17%) corresponds to indirect staff (health facility administrative personnel). In tertiary level facilities, medical doctors represented a lower portion of total staff costs compared to primary/secondary.

**Fig 5:**
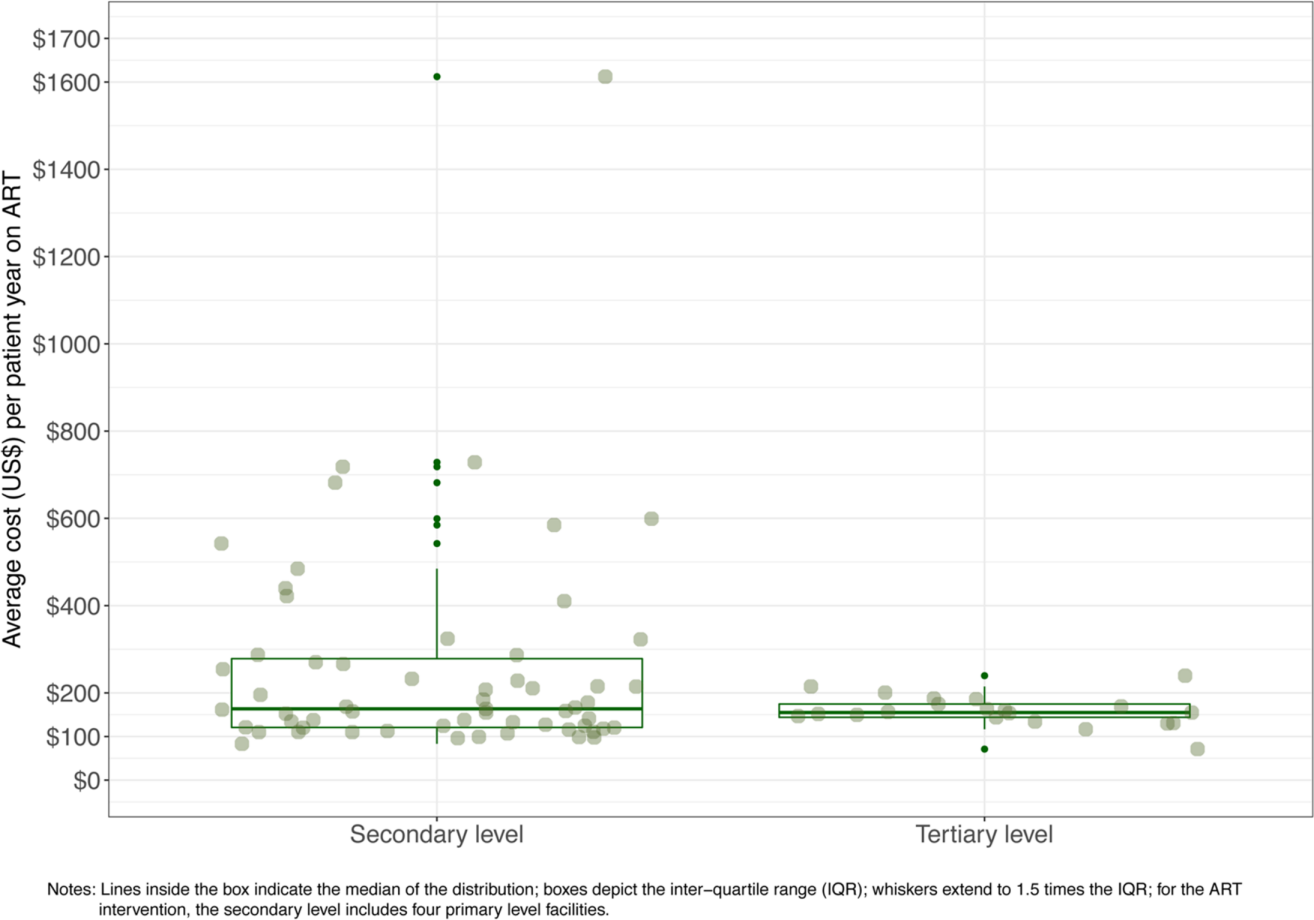
ART unit cost by facility type.

As illustrated in Fig 5, the average ART cost per patient varied by level of care: $257 in secondary-level facilities (including 4 primary-level facilities) and $159 in tertiary-level facilities. The figure also illustrates that the variability is larger in secondary facilities compared to tertiary-level facilities.

**Fig 6:**
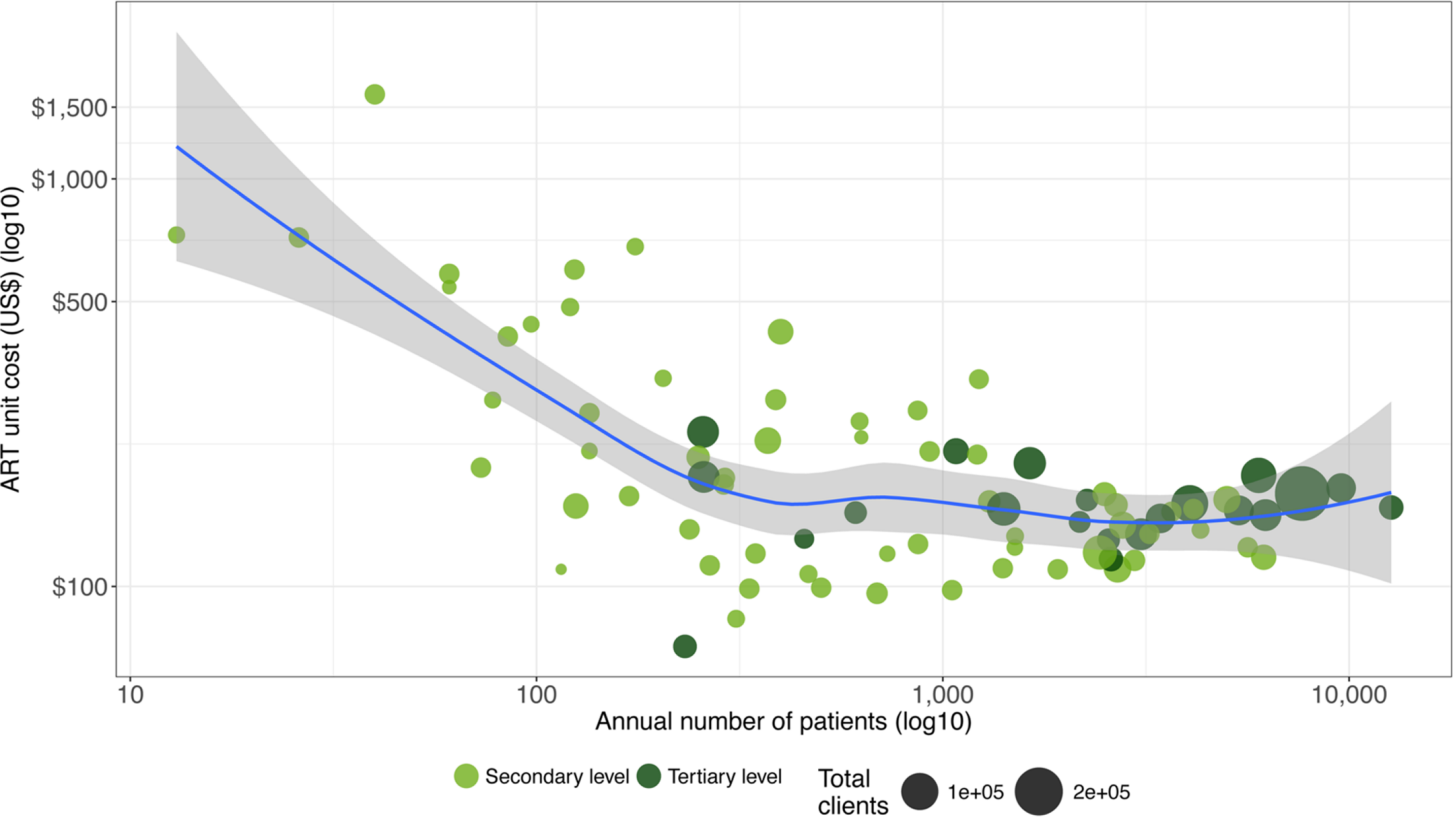
Correlation of unit cost and number of patients.

To explore the determinants of unit cost variation across facilities, we looked at the correlation between unit cost and the total number of patients treated (on a log-log scale), differentiating by level of care (primary/secondary and tertiary level). As Fig 6 illustrates, there is a nonlinear association between unit cost and number of patients: the unit cost decreases as facility size increases but at a decreasing rate. This correlation is stronger among secondary-level facilities. Tertiary level facilities treat a larger number of patients on average (3,503) compared to secondary level facilities (1,202), have lower unit costs, and demonstrate less variation with respect to scale.

Table 4 shows the results from the regression model estimating factors associated with the logarithm of the annual average costs per patient (unit cost) for the sample of facilities with information on provider´s vignettes and health providers´ experience variables (63 facilities) and the analytical sample of 80 facilities that excludes these two variables. According to the regression on the analytical sample (80 facilities), for secondary facilities, the annual number of patients is negatively associated with unit cost: a 10% increase in number of patients is associated with a 2.7% reduction in unit costs. However, unit costs in tertiary-level facilities are on average 179% lower compared to secondary level, but the association between unit costs and annual number of patients is less pronounced than in the case of secondary-level facilities; a 10% increase in patients is associated with only a 0.16% reduction in unit costs (this results from subtracting the coefficient for annual number of patients and the interaction term: -0.270 + 0.254 = -0.016). These results confirmed what we showed in the descriptive analysis (Fig 6): unit costs decrease significantly more with number of patients in secondary-level facilities than in tertiary-level facilities. With respect to task-shifting, facilities providing ART with nurses have 31.9% lower costs compared to facilities with doctors. None of the management variables were statistically significant in the regression model.

**Table 4:** Factors associated with the logarithm of the average annual ART cost/patient (unit cost).

Dimension	Variable	Coefficient		
**Supply**	Annual number of patients (ln)	-0.270** [0.047]	-0.251** [0.055]	
	Level of care (tertiary = 1)	-1.797* [0.652]	-2.115* [0.799]	
	Level of care*Annual number of patients (ln)	0.254** [0.085]	0.286* [0.102]	
**Process quality**	Competence		-0.334* [0.137]	
**Service delivery model**	Facility uses task shifting	-0.319** [0.097]	-0.283** [0.101]	
**Experience staff attending patients**	Proportion of staff with university degree or higher		0.094 [0.124]	
	Number of patients seen per day by clinical staff		-0.001 [0.001]	
	At least one staff with more than 10 years of experience in HIV		-0.007 [0.061]	
**Management**	Performance based incentives	0.163 [0.109]	0.063 [0.140]	
	Incentives for good performance	0.031 [0.100]	0.091 [0.120]	
	Sanctions for poor performance	-0.093 [0.098]	0.053 [0.140]	
	External supervisions received	0.153 [0.099]	0.125 [0.114]	
	Transparency	-0.059 [0.107]	-0.150 [0.136]	
	Community involvement	0.048 [0.126]	0.086 [0.391]	
**Constant**		7.048** [0.334]	6.930** [0.391]	
**Adjusted R- squared**		0.513	0.522	
**Observations**		80	63	

** Significant at 1%

* significant at 5%. Robust standard errors in brackets (White-Huber)

In the sample of 63 facilities with information from the provider´s vignettes, the results are similar, but we also see that facilities with competence scores greater than 80 have 28.5% lower costs compared to facilities with lower competence scores.

Results from the fully interacted model, which includes interactions between facility characteristics (scale, level of care and task-shifting) and the six binary management variables are presented in Table 5. The table shows the coefficients for each facility characteristic by management level: high or low. Overall, we can identify three distinct patterns across all management indicators. The first one is related to the association between scale and unit costs; the second relates to the differences in unit costs between secondary and tertiary facilities; and the third pattern is related to the association between task shifting and unit costs.

**Table 5:** Factors associated with the logarithm of the average annual ART cost per patient (unit cost) by management category (high/low distribution of management practices).

Variable	Performance based incentives	Incentives for good performance	Sanctions for poor performance	External supervisions	Transparency	Community involvement
**Annual number of patients (ln)**						
High	-0.408** [0.060]	-0.263* [0.115]	-0.262** [0.087]	-0.302**[0.049]	-0.202* [0.092]	-0.266+ [0.136]
Low	-0.218** [0.769]	-0.267** [0.056]	-0.272** [0.046]	-0.236* [0.104]	-0.287** [0.089]	-0.266** [0.063]
**Level of care**						
High	-6.061**[1.474]	-1.881 [1.141]	-0.429 [0.856]	-0.158 [0.546]	-1.950 [2.293]	0.517 [2.757]
Low	0.157 [0.716]	-1.256* [0.555]	-3.194** [1.097]	-2.410** [0.768]	-1.212 [0.842]	-1.952* [1.054]
Level of care*Annual number of patients (ln)						
High	0.926** [0.252]	0.269+ [0.155]	0.083 [0.122]	0.081 [0.080]	0.387 [0.359]	-0.090 [0.383]
Low	-0.009 [0.098]	0.210* [0.088]	0.486** [0.138]	0.340** [0.115]	0.169 [0.112]	0.315+ [0.166]
**Facility uses task shifting**						
High	-0.332 [0.220]	-0.182 [0.223]	-0.342* [0.168]	-0.293* [0.136]	-0.807** [0.256]	-0.741** [0.209]
Low	-0.387* [0.117]	-0.429**[0.124]	-0.431** [0.152]	-0.439** [0.154]	-0.228+ [0.117]	-0.266* [0.119]

Coefficients for each variable displayed by level of management were derived from the fully interacted model using margins in Stata 14.

** Significant at 1%

* significant at 5%

+ significant at 10%. Robust standard errors in brackets (White-Huber).

With respect to the association between scale and costs, in both groups of facilities there is a negative association between these two variables, consistent with economies of scale. However, in facilities with higher levels of incentives at the clinic level and with higher levels of supervision, the negative association is stronger.

We found that lower average costs and lower economies of scale are still found in tertiary-level facilities, as we found in the models presented in Table 4, however these associations are only found in facilities with lower levels of management practices across all management indicators (except for low performance-based incentives) and those with high incentives at the clinic level. Higher levels of management seem to reduce or even eliminate the differences between the two levels of care with respect to the scale effects.

Finally, the negative association between task shifting and unit costs is found in both groups of clinics except for high incentives (at the individual and clinic level). The association is higher in facilities with higher levels of transparency and community involvement.

## Discussion

We estimated unit costs of ART in Nigeria. The facility-level average was $231 USD, and the national average was $157. We found that, overall, staff and ARVs were the main components of the unit costs of ART services. Tertiary level facilities attend to more patients and therefore staff costs represent a lower percentage of the costs, while ARVs represent a higher portion.

The study showed wide variability in unit costs across facilities. The variation was associated with facility characteristics such as scale (number of patients): costs are lower as the number of patients increases, especially in secondary level facilities; and with level of care: unit costs are lower in tertiary level facilities.

In our view, finding lower unit costs in facilities serving more patients should not imply that provision be concentrated in larger facilities or in larger urban centers or localities, as it could have undesirable equity implications for populations living in more remote areas. Instead, our results suggest that through this type of analysis we can learn what makes small facilities with lower costs different from other comparably small facilities providing services more efficiently (at lower costs), and implement interventions or programs that ensure or at least facilitate this result. A second implication is that programs should be aware of the higher costs per patient that inevitably will be observed in smaller facilities and budget appropriately for that. Finally, in cases where it makes sense to merge small facilities without compromising access for the population, programs could consider doing so. Nigeria has recently expanded ART provision to primary level facilities making services more accessible. Future studies exploring more in-depth factors associated with efficiency could offer more evidence to improve efficiency in smaller units.

We also found that facilities where providers had competence scores above 80 had lower costs, suggesting that more competent providers may be more efficient at prescribing and monitoring patients, which could translate into savings.

Task shifting—shifting tasks traditionally performed by doctors to less specialized staff—resulted in lower costs compared to those facilities that include doctors attending to ART patients. Additionally, our second estimation controls for process quality (as measured by the clinical vignettes) and we found that even controlling for process quality, task shifting seems to be a good alternative to improve efficiency. Our study lacks outcome data to test whether task shifting compromises quality of care in Nigeria. However, previous literature suggests that in resource-limited settings, a strategy to reduce costs without compromising patients’ outcomes or to address the limited number of medical doctors in a country is to train nurses or other lower skilled personnel to attend to and prescribe ART treatment to HIV patients [16, 17]. Results from this study support this service delivery model and show that task shifting is associated with lower unit costs. This result is also consistent with a study conducted in South Africa that showed that costs of patients treated in a nurse-managed primary health care facility were lower than those of similar patients treated in hospital-based ART clinic, yet outcomes in both groups were similar [18].

Our results from the analysis of management indicators suggest that management does interact with facility characteristics and their relationship with unit costs. Specifically, our results suggest that management practices potentiate or thwart economies of scale, i.e. the reduction in costs accomplished by increasing the volume of services. For example, higher levels of incentives—at the facility level—and higher levels of supervision are correlated with higher reductions in unit costs, compared with lower levels of such practices. Incentives and supervision may motivate providers to create ways to use resources more efficiently and thereby reduce costs at any given volume of services.

Similarly, our results on level of care in different management contexts suggest that while tertiary facilities naturally can offer services at a lower average cost given their advantageous position as larger, urban, and more sophisticated sites, when higher levels of management practices are implemented, the difference between secondary and tertiary levels seem to fade. This implies that when smaller, less sophisticated sites implement good management practices, their performance—at least in terms of efficiency—is not dissimilar to tertiary facilities. This result adds to previous work which demonstrates that improved management practices can positively affect productivity and performance, both in production [19, 20] and in healthcare [21, 22].

With respect to task-shifting, we also found that facilities where higher levels of transparency and community involvement were implemented, the efficiency benefits of shifting the provision of care from medical doctors to nurses seem to increase. This result is consistent through all management indicators. Numerous studies show that task shifting can have this cost-saving effect [23, 24]. There is also some evidence that the synergistic interaction between supervision/mentorship and task shifting can produce facility-level performance improvement [25]. This is the first study to the authors’ knowledge to examine this interaction as it relates to efficiency in the public sector.

While our results cannot establish causal effects, given the dearth of evidence on the relationship between management practices and efficiency in public or non-for-profit health services, at the very least they suggest potentially fruitful hypotheses to test in experimental contexts.

The ART annual cost of $231 USD estimated in this paper is similar to the mean cost of $209 USD from a study conducted in 2012 in two primary and seven secondary level facilities in Nigeria [7] and similar to a $208 USD cost that was estimated at the facility level in four countries in Africa (Ethiopia, Malawi, Rwanda and Zambia) [26] and to the annual cost of $265 USD in a study conducted in Ethiopia. The first study also found that ARVs and personnel were the largest components of total ART costs, consistent with our results. In contrast, unit costs were considerably lower compared to the cost per patient of $742 USD estimated in a study conducted in 2004 [8]. Potential reasons for the difference between our results and this study are: the cited study used information from only 5 treatment hospitals—implying that the sample did not capture enough variation for more accurate estimates of unit costs variation at the country level—and ARV costs in 2004 were higher ($368) than in 2013—the year of our study. Additionally, laboratory monitoring tests ($169) that included full blood count, urea, creatinine, blood sugar, liver function tests, and viral loads were included in the 2004 study whereas our study included only CD4 as laboratory tests.

Some limitations should be considered when interpreting our results. Our estimates of ARV and TB drugs costs rely on incomplete, facility-level records on drug inventories, instead of clinical records of individual patients. Estimation of ARVs costs was not entirely based on micro-costing methods—*i*.*e*. from data at the level of utilization—due to incomplete regimens reported. However, our estimation of ARVs is based on facility-level data on the different drugs dispensed. Therefore, as prices of treatment are constant for the entire sample, the analysis captures real variability in prescription practices across facilities. Our costs estimations do not include costs associated with opportunistic infections aside from TB treatment. However, according to other studies, these costs represent a smaller portion of total costs [27]. Finally, we acknowledge that from a sample of 147 facilities offering ART services, we were only able to study 80 facilities with adequate information on patients and ARVs (facilities that had at least six months of information on ARV drugs and number of patients). As the analytical sample had more tertiary level facilities and fewer primary, our cost estimations may be downward biased because we found lower unit costs in tertiary level facilities.

We acknowledge that measuring time allocation using data collected retrospectively (last week of work) could potentially result in recall bias and also in overestimation of time allocated to specific activities. We tried to minimize these potential sources of bias in two ways: by asking activities during the previous week instead of a period too far back in the past or an average during the previous month or year; and by asking providers to report time spent on specific daily activities and times each day of the previous week.

Facilities excluded from the analytical sample due to missing information were primary- and secondary-level facilities. We acknowledge that this has implications on the representativeness of the sample. We found that a higher percentage of the excluded facilities had been offering ART services for less than 12 months. Because the ART program in Nigeria had recently expanded, some of the excluded facilities may have had fewer patients at the time of data collection and also incipient systems to monitor indicators. In this sense, our analytical sample is representative of more established facilities. In order to explore potential implications of this relatively large loss of observations, we conducted a sensibility analysis in which we explored the association between costs and maturity of the ART programs at the facility-level by running the regression that explores factors associated with unit costs for facilities with more than 12, 24 and 36 months operating. We found similar results compared to results in Table 4 suggesting that maturity did not bias our findings (S3 Table). We also tested the regression model adding binary variables for facilities operating for 12, 24 and 36 but none of the variables were statistically significant.

We acknowledge that we only measured performance based on vignettes directed towards medical doctors and missed information on other lower skilled staff attending to patients. In this sense, the process quality indicator we are capturing in our study refers to the most skilled staff in facilities, which is expected to be correlated with other less skilled staff performance.

Finally, we acknowledge that some of the variability in costs may be driven by geographic characteristics, such as urban/rural areas, that we were not able to explore in the study.

In spite of these limitations, this is the first study conducted in Nigeria to assess costs using data collected at the facility level in a significantly larger sample than previous studies. In any study, estimations of unit costs impose the challenge of interpreting and comparing the results. Estimation of average cost per patient-year on ART in Africa varies widely mainly due to differences in methods, scope and settings [3, 26, 28–34]. In terms of methods, studies can vary in the elements of the service provision included in the measurement of cost (staff, drugs, laboratory tests, capital, utilities, training, supervision, etc.). Some studies collected data on health care utilization at the facility level to estimate costs (patient charts, electronic data sets, pharmacy and other records), whereas others use a normative approach based on guidelines, or a combination of both. The scope can also differ across studies: some include costs for pre-ART and ART patients while others exclude pre-ART. Some cost only new patients (first year of treatment), whereas other include all active patients in a specific period (retrospectively or in cohorts of HIV patients). The settings studied are also heterogeneous, not only in terms of countries, but even within countries; there are studies that include a sample of patients within a single hospital, while others include a sample of different types and levels of facilities. On top of all these differences, the standards for reporting results also vary widely across costs studies. Our study aims to provide a reference point by reporting extensively on the methods used, including the largest sample size thus far in a costing study in Nigeria, and relying on micro-costing methods as much as possible.

Results from this study can help to inform further research that comprehensively evaluates the characteristics that make facilities more efficient than other comparable facilities (with similar number of patients or same level of care). Based on our results, interventions to improve competence and strategies to promote task shifting may be promising as means to increase efficiency. Finally, our results suggest that management practices interact with service delivery characteristics in ways that can potentially increase or reduce unit costs. Further investigation on this topic is necessary as health systems need more information on determinants of efficiency.

## Supporting information

S1 Table Distribution of time of ART service provision in the analytical and the excluded sample by level of care. (DOCX)

S2 Table Questions included in the management dimensions (additive scores*). (DOCX)

S3 Table Factors associated with the logarithm of the average annual ART cost/patient (unit cost) by time of ART provision. (DOCX)
