# Supplementary material for: Explaining the heterogeneity in average costs per HIV/AIDS patient in Nigeria: The role of supply-side and service delivery characteristics
Source: PLoS One. 2018 May 2;13(5):e0194305. doi: 10.1371/journal.pone.0194305 (PMC5931468; doi:10.1371/journal.pone.0194305)
Supplement: S1 Table — (DOCX) [file pone.0194305.s001.docx]

**Supporting information**

**Table S1- Distribution of time of ART service provision in the analytical and the excluded sample by level of care**

| **Time ART service provision** | **Level** | **Analytical Sample** | **Excluded sample** | **Total** |
| --- | --- | --- | --- | --- |
| Less than 12 months | Primary | 2 (50.0%) | 9 (81.8%) | 11 |
|  | Secondary | 3 (5.0%) | 14 (28.5%) | 17 |
|  | Tertiary  **Total** | 1 (4.7%)  **6 (7.5%)** | 1 (14.2%)  **24 (35.8%)** | 2  **30** |
| More than 12 months | Primary | 2 (50.0%) | 2 (18.2%) | 4 |
|  | Secondary | 52 (95-0%) | 35 (71.5%) | 87 |
|  | Tertiary | 20 (95.3%) | 6 (85.8%) | 26 |
|  | **Total** | **74 (92.5%)** | **43 (64.2%)** | **117** |
| **Total** |  | **80** | **67** | **147** |

In primary level, 81.8% of the facilities excluded had less than 12 months offering services compared to 50% in the analytical sample. For secondary level 28.5% in the excluded sample vs 5% in the analytical sample had less than time offering ART services. In tertiary level facilities the percentages were 14.2% vs 4.7%.
