# Supplementary material for: Explaining the heterogeneity in average costs per HIV/AIDS patient in Nigeria: The role of supply-side and service delivery characteristics
Source: PLoS One. 2018 May 2;13(5):e0194305. doi: 10.1371/journal.pone.0194305 (PMC5931468; doi:10.1371/journal.pone.0194305)
Supplement: S2 Table — (DOCX) [file pone.0194305.s002.docx]

**Supporting information**

**Table S2- Questions included in the management dimensions (additive scores*)**

| **Management dimension** | **Questions** |
| --- | --- |
| Performance-based incentives | Is funding based on number of clients served?  Is funding based on number of services delivered?  Is funding based on amount of inputs used? Is funding based on quality of service? Is funding based on levels of drugs and supplies (i.e. no stock outs) |
| Incentives for good performance | Can the staff at this facility receive rewards for high or  improved performance?  Does the staff ever receive extra payment/bonuses for good performance  Does the staff ever receive time off for good performance Does the staff ever receive verbal recognition for good performance  Does the staff ever receive preferred rotation for good performance  Does the staff ever receive certificates for good performance  Does the staff ever receive training for good performance  Do staff receive [commodities] for good performance? |
| Sanctions for poor performance | Does the facility warn or apply sanctions to its staff for:  Unprofessional behavior  Nor performing responsibilities  Absence without cause  Persistent tardiness  Persistent absenteeism  Impoliteness to patients  Impoliteness to other health workers  Impoliteness to supervisors  Persistent rudeness  Receiving many patient complaints  Theft or misappropriation |
| External supervisions | Has the HIV unit at this facility received supervisory visits from the Ministry of Health, the state-level health office, or the LGA-level health office, and/or implementing  partners in the entire year 2013?  Did the supervisor [. . . ] during the visit?: Observe or examine the quality of HIV service delivery at the facility based on federal guidelines, standard operating procedures, or policy  Conduct an audit of patient cards  Check HIV service complaints  Inquire about HIV facility records or registers  Discuss general implementation problems and challenges  Meet with staff working in HIV services  Meet with community health workers and/or volunteers involved in HIV services  Other supervision actions |
| Transparency | Does the facility inform the community about the performance of HIV prevention services?  Which strategies are used to report the levels of performance?  In 2013, did the facility have more than 3 meetings organized with the community to report the performance of ART services?  In 2013, did the health facility release information on HIV prevention and treatment outcome indicators to the community for ART more than 8 times? |
| Community involvement | Is there a governing board for this facility?  Does the governing board meet monthly or more frequently?  Since 2013, has the council [. . . ]?  Bought items or awarded personnel bonuses from available funds  Communicated patient complaints or expressions of gratitude to the facility  Monitored the delivery of HIV-related drugs to the facility  Monitored the delivery of HIV-related supplies to the facility  Participated in discussions about priorities at the facility  Participated in discussions about allocations for the various services  Other  Does a ward development committee / facility board exist for this facility? Does the community have a role in monitoring and providing  feedback to improve the performance of the facility? Does this facility involve community groups in providing health service provision to the community? |

*To validate the management scores, we used Cronbach´s alpha values as a measure of internal consistency: scores with alpha values lower than 80 were not included.
