# Supplementary material for: Explaining the heterogeneity in average costs per HIV/AIDS patient in Nigeria: The role of supply-side and service delivery characteristics
Source: PLoS One. 2018 May 2;13(5):e0194305. doi: 10.1371/journal.pone.0194305 (PMC5931468; doi:10.1371/journal.pone.0194305)
Supplement: S3 Table — (DOCX) [file pone.0194305.s003.docx]

**Supporting information**

**Table S3- Factors associated with the logarithm of the average annual ART cost/patient (unit cost) by time of ART provision**

| **Variable** | **More than 12 months** | **More than 24 months** | **More than 36 monhts** |
| --- | --- | --- | --- |
| Annual number of patients (ln) | -0.280 [0.055]** | -0.227 [0.065]** | -0.200 [0.081]* |
| Level of care (tertiary=1) | -1.441 [0.494]** | -1.253 [0.655]* | -0.923 [0.820] |
| Level of care*Annual number of patients (ln) | 0.214 [0.069]** | 0.182 [0.088]* | 0.138 [0.109] |
| Facility uses task shifting | -0.252 [0.100]* | -0.201 [0.110]+ | -0.229 [0.114]* |
| Performance based incentives | 0.210 [0.107]+ | 0.165 [0.122] | 0.161 [0.129] |
| Incentives for good performance | 0.006 [0.103] | -0.067 [0.090] | -0.099 [0.094] |
| Sanctions for poor performance | -0.097 [0.099] | -0.158 [0.100] | -0.196 [0.104] |
| External supervisions received | 0.137 [0.100] | 0.136 [0.091] | 0.084 [0.100] |
| Transparency | -0.051 [0.105] | -0.099 [0.110] | -0.045 [0.127] |
| Community involvement | 0.112 [0.125] | 0.194 [0.134] | 0.208 [0.136] |
| Constant | 7.056 [0.399] | 6.729 [0.452] | 6.386 [0.569] |
| Observations | 74 | 59 | 55 |

** Significant at 1%, * significant at 5%. + significant at 10%. Robust standard errors in brackets (White-Huber)
